# Instrument-Independent CAD Spectral Databases: Absolute Cross-Section Measurements In QQQ Instruments

**DOI:** 10.6028/jres.092.019

**Published:** 1987-06-01

**Authors:** Richard I. Martinez, Seksan Dheandhanoo

**Affiliations:** National Bureau of Standards Gaithersburg, MD 20899

**Keywords:** calibration, cross sections, tandem mass spectrometry, target thickness

## Abstract

The energy dependence of the cross section, *σ*(*E*), for the symmetric (resonant) charge transfer reaction Ar^+^(Ar,Ar)Ar^+^ was measured in our triple quadrupole (QQQ) tandem mass spectrometer.^1^ Our *σ*(*E*), for *P*≃0.04–0.43 mtorr and *E*≃5 – 60 eV (LAB) [the range of collision energies used for collisionally activated dissociation (CAD)], agrees to within 10% with the Rapp-Francis theory (impact parameter method in the two-state approximation), as corrected by Dewangan. We measured identical *σ*(*E*) from both the rate of reactant ion decay and the rate of product ion formation; i.e., our instrument is kinetically well behaved. The measurement of these *σ*(*E*) in other QQQ instruments can be used to validate whether or not a QQQ instrument has been properly designed to be kinetically well behaved. This is essential if generic, instrument-independent CAD spectral databases are to be developed on the basis of the absolute cross sections for the CAD of *known* ionic substructures. That is, since tandem mass spectrometry (MS/MS) exploits the ion fragmentation patterns characteristic of ionic substructures, the characteristic profiles [“breakdown curves”] of ion abundance versus target thickness (or collision energy) correspond uniquely to the sequence: (parent)*_i_* $\underset{\to }{\sigma_{ij}}$ (daughter), $\underset{\to }{\sigma_{jk}}$ (granddaughter)*_k_*, etc. Hence, computer simulation of experimentally observed breakdown curves enables the structure of an *unknown* species to be assigned on the basis of the absolute cross sections *σ_ij_, σ_jk_*, etc. for CAD of *known* ionic substructures *i*, *j*, *k*, etc. Thus, if the calculated and experimental breakdown curves agree, the structure would be characterized.

## Introduction

Triple quadrupole (QQQ) tandem mass spectrometry (MS/MS) is an analytical tool which can be used for rapid, direct speciation of complex multicomponent mixtures [1].^2^ The analysis makes use of the collisionally-activated dissociation (CAD) of “parent” ions.^3^ A “parent” ion selected by the first quadrupole (Q1) is interacted with a target gas within the second quadrupole (Q2). Q2 channels undissociated “parent” ions and “progeny” fragment ions into the third quadrupole (Q3) for mass analysis. The instrument thus produces a CAD spectrum of each initially-selected “parent” ion.

In principle, standard CAD spectra of a variety of ions (fragment ions, molecular ions, protonated molecules, etc.) could be generated and collected as reference libraries, to be used for comparison against unknown spectra in a manner analogous to the use of reference libraries in the data handling systems of ordinary electron impact mass spectrometry. Further, it should be possible to infer the identity of an unknown complex molecule by identifying the ionic substructures of fragment ions generated in its CAD spectrum. However, to date reference libraries of CAD spectra have not been collected because of a lack of standardization of operating conditions of such instruments [2].

There are several instrument parameters which can cause significantly different CAD spectra to be observed for any given molecule. The key parameters are: 1) the number of collisions undergone by a “parent” ion within Q2, a parameter usually characterized in terms of “target thickness,” which is defined as [(actual path length traversed by the ion through the gas target) × (effective number density of the CAD target gas)]; 2) the duration of the interaction between the “parent” ion and the target gas, which is determined by the collision energy for “parent” ions entering Q2; and 3) the energy level of the analyzing quadrupole Q3 relative to that of Q2 which, because of the translational energy distribution of the “progeny” ions, determines whether or not some progeny ions can enter Q3.

Results of a recent international round robin [2] demonstrated that the target thickness is not a wellcontrolled parameter, with estimated target thicknesses differing by factors of 2–4 from apparent actual values. The problem of determining target thickness is complicated in QQQ instruments because of the complex oscillatory trajectories of ions within a quadrupole mass filter [3–7]; the actual path length traversed by the ion through the CAD gas can be significantly longer than the nominal gas target length [6]. Moreover, in QQQ instruments utilizing a molecular beam target (Type A configuration [2]) the problem is further complicated because of a lack of information about the extent of overlap of the projectile ion beam and the molecular beam target. On the other hand, in QQQ instruments utilizing a collision chamber (Type B configuration [2]), the actual target thickness can be significantly greater than an estimated value based on the length of the Q2 collision chamber and the pressure within it if the gas plume extends beyond the confines of the Q2 collision chamber into Q1 and Q3.

### Kinetic Method

In a recent study from this laboratory [8] it was suggested that these problems can be circumvented by using a kinetic method to measure the effective target thickness within a QQQ instrument. That is, if a reaction can be identified for which the cross section (or rate coefficient) is well established as a function of collision energy, then a simple measurement of the intensity of the reactant ion and/or product ion in the absence and presence of CAD target gas at known collision energy leads to an experimental determination of the target thickness. For example, for the charge transfer reaction:

$$A^{+}+B\to B^{+}+A$$ (1)

under pseudo-first order conditions ([*B*]>> [*A*^+^]),^4^

$$Ln\;Y\equiv Ln{\left[A^{+}\right]}_{0}/[A^{+}]=\sigma_{E}L_{\text{eff}}[B]\equiv \beta \;P_{B}$$ (2)

where *σ_E_* is the value of the reaction cross section at a collision energy *E, L*_eff_ = effective path length of the oscillatory trajectory traversed by a projectile ion through the CAD target gas, *L*_eff_ [*B*] = effective target thickness for A^+^ in B, *β*=proportionality constant, and *P_B_* = pressure of target gas B corresponding to [*B*]. Hence, in the absence of other loss processes for A^+^, measurement of *Ln* [*A*^+^]_0_/[*A*^+^] provides in-situ calibration of the effective target thickness if *σ_E_* is known. Moreover, if there are no other production processes for B^+^, if there is no mass discrimination within the QQQ mass filters between the m/z of A^+^ and the m/z of B^+^, and if the ion collection efficiency approaches 100%, then [*B^+^*]*_∞_* = [*A*^+^]_0_, and

$$Ln\;W=\sigma_{E}L_{\text{eff}}[B]\equiv \beta \;P_{B}$$ (3)

where *W*=[*B^+^*]_∞_/{[*B^+^*]_∞_ − [*B*^+^]}≡[*A*^+^]_0_/[{*A*^+^]_0_–[*B*^+^]}. Hence, obtaining the same result from reactant ion loss and product ion formation experiments (i.e., *Ln Y* and *Ln W* measurements, respectively) provides strong assurance that a QQQ instrument is kinetically well behaved. That is, it provides a very important test that the instrument parameters and the reaction kinetics are well controlled (no back reactions, no impurity reactions, no scattering losses, no fringing fields, well- confined gas target, etc.).

In our earlier study [8], the symmetric (resonant) charge transfer reaction Ne^+^(Ne,Ne)Ne^+^ was used as a calibrating reaction for the validation of the target thickness measurements in our QQQ instrument. Abundant experimental and theoretical results had been previously reported for this reaction. Furthermore, because the NBS instrument had been constructed to incorporate the design considerations detailed by Dawson and coworkers [3–7], eq (4) [6] could be used to estimate *L*_eff_*=R L*_actual_.

$$R={\left[1+(0.0738{r_{0}}^{2}F^{2}M/E)\right]}^{0.5}.$$ (4)

Here *L*_actual_ is the actual rectilinear pathlength for a well-confined CAD gas target; *M* = mass of projectile ion (in amu), *E* = axial ion energy (in eV), *r*_0_=field radius (in cm), *F* = rf frequency (in MHz). Equation (4) is based on operation of Q2 with the Mathieu parameters [3,4] at *a*_2_=0, *q*_2_=0.28 [6],^5^ It was shown [8] that when the effective target thickness was estimated by using eq (4), values for the absolute reaction cross section derived from eq [2] were in excellent agreement with theoretical predictions, as well as with previous experimentally- determined values. Furthermore, identical values for the reaction cross section were derived from reactant ion loss [eq (2)] and product ion formation [eq (3)] experiments, thus confirming that the NBS instrument is kinetically well behaved.

This paper reports results of an analogous exercise carried out using the ^40^Ar^+^(^x^Ar,^40^Ar)^x^Ar^+^ reaction^6^ for *Ln Y* measurements and the ^36^Ar^+^ (^40^Ar,^36^Ar)^40^Ar^+^ reaction for *Ln W* measurements.^6^ The Ar^+^(Ar,Ar)Ar^+^ reaction is of special interest because argon is a target gas commonly used for CAD. Thus, this reaction may provide a convenient calibrant species for target thickness determinations in other laboratories. Since reference spectra for CAD libraries can be utilized only if they were obtained under conditions such that the target thickness is specified, the results reported here may permit the easy standardization of operating conditions for the determination of such reference spectra.

## Experimental

**Figure 1:**
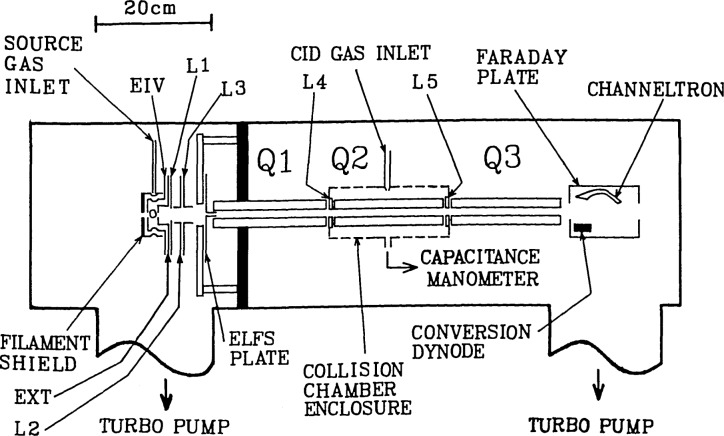
Schematic of QQQ instrument. EIV, EXT, L1-L5, etc. are ion optics lens elements; ELFS™ and CHANNELTRON™ are registered trademarks of Extrel and Galileo Electro-Optics, respectively.

Our specially designed QQQ instrument can be configured to use either a molecular beam (Type A) or collision chamber (Type B) configuration (see schematic, fig. 1). All experiments reported here utilized the Type B configuration.

An abbreviated description of the instrument follows (a detailed description will be published elsewhere [10]). The instrument was manufactured by Extrel, Inc.^7^ to conform to the design considerations stipulated by Dawson and coworkers [3–7]. It consists of three standard 7-270-9 quadrupole rod assemblies (Q1, Q2, Q3) mounted in tandem on a special multipurpose track. Each mass filter assembly is operated at 1.2 MHz, controlled by a 300-watt Model 150-QC quadrupole power supply and associated quadrupole control electronics. A C-50-IC controller regulates the standard Extrel electron impact ionizer mounted on the differential pumping wall. This ionizer has a filament perpendicular to the cylindrical quadrupole axis and has been modified to accommodate crossed molecular and laser beams. Each QQQ system parameter is computer controlled via its respective 16-bit DAC by the standard 8086-based Extrel Triple Quad Data System used for instrument control and data acquisition.

For the Type B configuration, Q2 is surrounded by a collision chamber enclosure while Q1 and Q3 are completely nude (no housing), and are adequately pumped by four 1200 1/s turbomolecular pumps, ensuring a well-confined collision region. The actual length of the collision region from the front face of the L4 aperture to the rear face of the L5 aperture is *L*_actual_=21.74_5_±0.07_5_ cm. All kinetic measurements were based on operation of Q2 with the Mathieu parameters [3,4] at *a*_2_=0, *q*_2_= 0.28 [6]. For our instrument, *r*_0_=field radius=0.684 cm (quadrupole rod diameter = 1.59 cm), *F* = rf frequency =1.2 MHz, and the *R* correction factor from eq (4) is ca. 1.02 at *E* =60 eV and 1.18 at *E* = 5 eV. Furthermore, the diameter of our L4 and L5 inter-quadrupole lens apertures is 1.27±0.025 cm {> 1.4*r*_0_ [6]}, and thus conforms to the requirements for closely-coupled quadrupole fields [6]. Pressure measurements in the center of the collision chamber were made with a 1 torr MKS 310CA Baratron capacitance manometer [appropriate corrections were made for thermal transpiration (≃3%) etc.].

**Figure 2:**
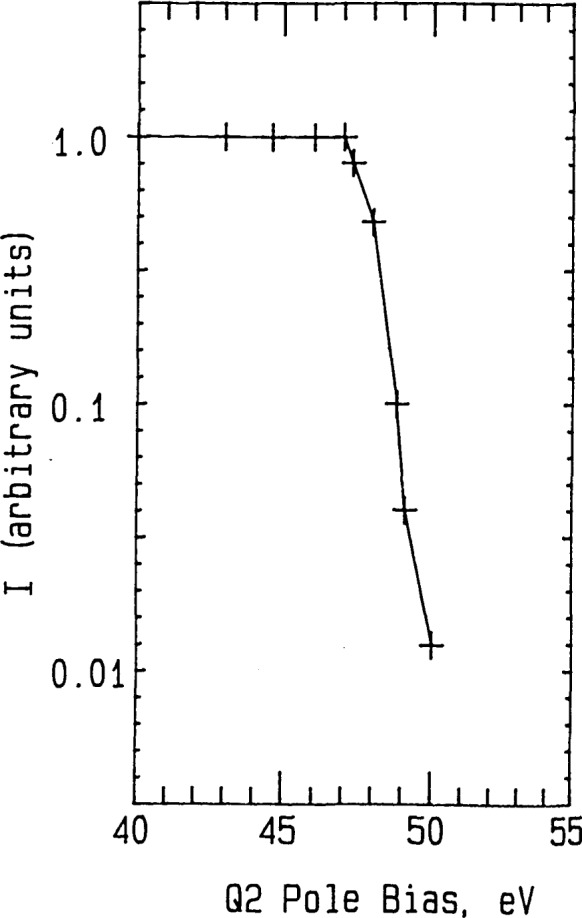
Energy distribution of Ar^+^ projectiles entering Q2. *I* = Ion current in arbitrary units.

Ar^+^ ions were generated by 70 eV electron impact [11], and the Ar^+^ projectiles were selected by Q1 [19]. The energy spread of the projectiles entering Q2 was determined to be ⩽1.8 eV for 90% of the ions [⩽3 eV for 99% of the ions] when measured by using the Q2 pole bias (rod offset) to generate a stopping potential curve (see fig. 2). *E*_90%_ is the Q2 potential required to stop 90% of the ions. The collision energy *E*_coll_ was selected by setting the Q2 pole bias = *E*_90%_ − *E*_coll_.

**Figure 3:**
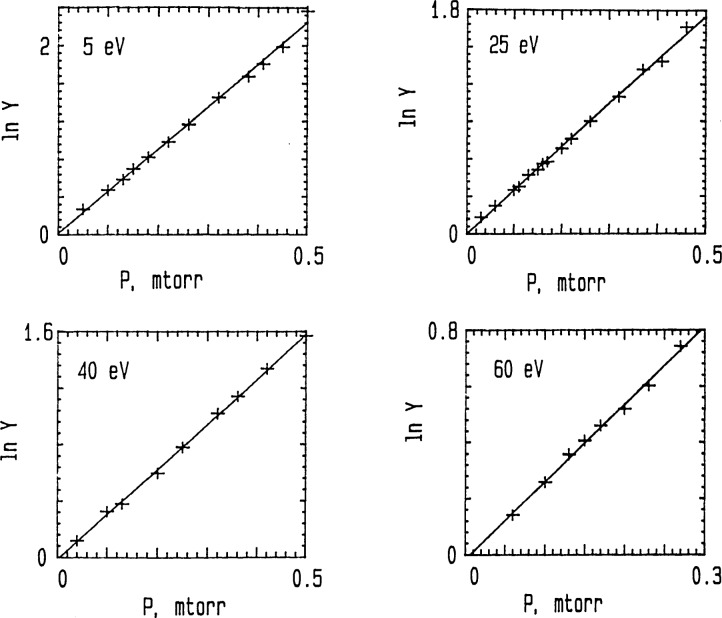
Projectile ion decay experiments. Plots of *Ln Y* versus Ar target pressure *P* at fixed values of *E*_coll_. *Y*=[^40^*Ar^+^*]_0_/[^40^*Ar^+^*] and *Ln Y=k_E_ L*_eff_ (m/2*E*_coll_)^1/2^ [*Ar*] *= σ L*_eff_ [*Ar*]≡*β P, L*_eff_=effective path length traversed by ion within Q2 collision chamber (corrected for rf effects [6]). *m* = mass of ^40^Ar^+^ projectile.

**Figure 4:**
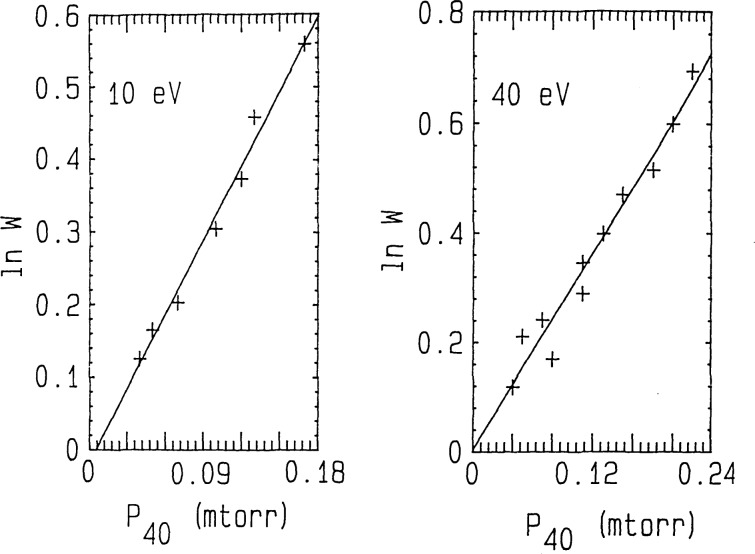
Product growth experiments. Plots of *Ln W* versus *P*_40_ at fixed values of *E*_coll_. $W=\frac{{\left[{\;}^{36}Ar^{+}\right]}_{0}}{\left\{{\left[{\;}^{36}Ar^{+}\right]}_{0}-\left[{\;}^{40}Ar^{+}\right]\right\}}$ and *Ln W=β P*_40_ where *β* is same as that of figure 3 and *P*_40_ is partial pressure of ^40^Ar.

Projectile decay experiments (cf. fig. 3) were performed at each selected collision energy by setting the Q3 pole bias more positive relative to the Q2 pole bias (e.g., Q3−Q2≃3 to 40 V for *E*_coll_*≃*5 to 60 eV) to ensure only *unreacted* projectiles were able to enter Q3 [25]. Product growth experiments (cf. fig. 4) were performed by setting the Q3 pole bias sufficiently negative relative to the Q2 pole bias (e.g., Q2−Q3≃110 to 140 V for *E*_coll_≃40 to 10 eV) to ensure that all ions (products *and* unreacted projectiles) were drawn out of Q2 into Q3 [25]. The typical ion collection efficiency is ⩾97%; i.e., the total ion current for products+unreacted projectiles (i.e., with CAD gas on) ⩾97% of the initial projectile ion current (i.e., with CAD gas off). This high ion collection efficiency allows one to set [^40^Ar^+^]_∞_ = [^36^Ar^+^]_0_ when used in *Ln W* [as defined in eq (3) and in fig. 4]. For both types of experimental measurements [viz., projectile ion decay (i.e., *Ln Y* measurements) and product ion growth (i.e., *Ln W* measurements)], several CAD target gas pressures were used (see figs. 3 and 4).

## Results

Figures 3 and 4 show typical data for projectile ion decay and product ion growth experiments, respectively. Here *P* is the total Ar target gas pressure (*P*=*P*_40_+*P*_38_+*P*_36_; where *P*_40_, *P*_38_, and *P*_36_ are, respectively, the partial pressures of ^40^Ar, ^38^Ar, and ^36^Ar). The well-established isotopic abundance of ^40^Ar(99.6003±0.0006% ^40^Ar; 0.0632±0.0001% ^38^Ar; 0.3365±0.0006% ^36^Ar [9]) was used to determine *P*40 from the measured *P.*

**Figure 5:**
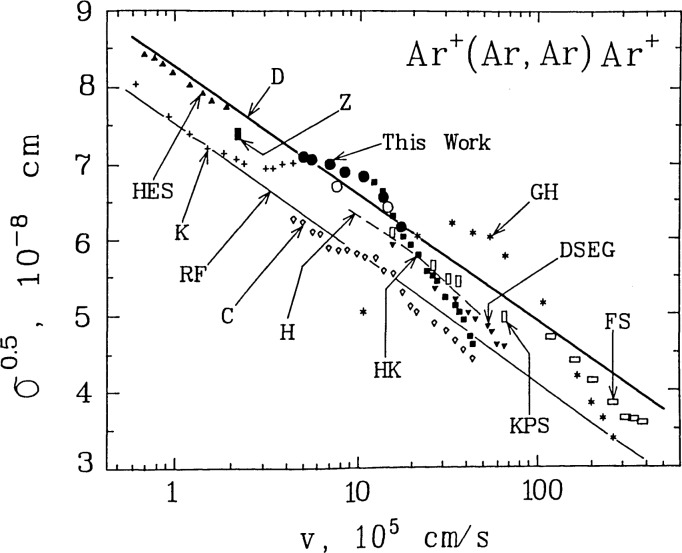
Plot of *σ*^1/2^ (reaction cross section)^1/2^ versus projectile ion velocity v. Comparison of our results with other workers and with theoretical models. ●—our projectile decay experiments, ○—our product growth experiments. Label [reference]: Z [28], DSEG [29], GH [30], C [31], K [32], HK [33], HES [34], H [35], FS [36], KPS [37], RF [42], D [43].

Figure 5 shows the energy dependent cross sections for Ar^+^(Ar,Ar)Ar^+^ in the format commonly used for resonant charge transfer reactions; viz. σ_0.5_ vs. *Ln* v, where v is the projectile ion velocity. For *E*_coll_≃5–60 eV [corresponds to v≃0.5–1.8 (×10^6^) cm s^−1^], the *σ*(*E*) shown as (●) in figure 5 were derived from *Ln Y* vs. *P* measurements for the ^40^Ar^+^ projectile ion reacting with ^40^Ar+^38^Ar + ^36^Ar in the target gas (see fig. 3). For *E*_coll_=10 and 40 eV, *Ln W* vs. *P*_40_ measurements of the rate of production of ^40^Ar^+^ in ^36^Ar^+^(^40^Ar,^36^Ar)^40^Ar^+^ {see fig. 4} led to the *σ*(*E*) shown in figure 5 as (○). These were substantially the same as the *σ*(*E*) determined from the *Ln Y* vs. *P* measurements for the ^40^Ar^+^ projectile.

## Discussion

Together with our results for Ar^+^(Ar,Ar)Ar^+^, figure 5 also summarizes experimental [28–41] and theoretical [42–47] results for this reaction from the literature [48]. Prior to our work it was not clear which theoretical model one could or should use to obtain reliable estimates of *σ_E_* values for use in target thickness calibrations in the 5–60 eV range of collision energies, the range typically used for CAD experiments. The results reported here for *σ*(*E*) (see fig. 5) are in excellent agreement with the *σ*(*E*) predicted by the Rapp-Francis theory (impact parameter method in the two-state approximation) [42] as corrected by Dewangan [43] (solid line D in fig. 5), as well as with the experimental *σ*(*E*) of other workers (see fig. 5, data labeled HES [34], Z [28], H [35], KPS [37], DSEG [29], FS [36]). For the data labeled C [31], the *σ_E_* values are significantly lower than those of the Dewangan line (labeled D) [43] and of other workers; however, the slope of his *σ_E_* vs. *E* plot shows substantially the same *σ*(*E*) as that of the Dewangan line. On the other hand, the *σ*(*E*) of the data labeled HK [33] clearly differs from that of the Dewangan line and of other workers, even though some of the *σ_E_* values labeled HK overlap some of the *σ_E_* values of other workers. Hence, the data of figure 5 labeled C [31] and HK [33] are not considered further.

Our results show excellent agreement between the *σ_E_* values derived from *Ln Y* measurements (reactant ion loss; ● in fig. 5) and the corresponding values derived from *Ln W* measurements (product ion formation; ○ in fig. 5). This concordance establishes 1) that our instrument is kinetically well behaved, and 2) the validity of Dawson’s design considerations (closely-coupled quadrupole fields, properly filled acceptance, etc.) [3–7]. Similar agreement between *Ln Y* and *Ln W* measurements has also been observed in our Type B configuration for Ne^+^(Ne,Ne)Ne^+^ [8] and Ar^+^(N_2_,Ar)N_2_^+^ [49], further confirming that our instrument is kinetically well behaved. Thus we can use the *σ_E_* values measured in our Type B configuration to determine the effective target thickness of Ar in our Type A configuration. However, similar performance is expected only in kinetically well behaved QQQ instruments which incorporate Dawson’s design considerations [3–7].

## Conclusions

The *kinetic method* described in the *introduction* potentially can provide a means whereby absolute target thicknesses for any gas can be accurately calibrated in-situ in kinetically well behaved QQQ instruments (in Type A or Type B configurations) for collision energies in the 5–60 eV range. Moreover, since the *σ_E_* values for Ar^+^(Ar,Ar)Ar^+^ are not strongly dependent on *E* over the range of interest for CAD experiments {*σ*_5eV_≃1.3 *σ*_60eV_), the kinetic method should provide fairly accurate target thickness calibrations even if the projectile energy distribution in other QQQ instruments is not as narrow as in the NBS instrument.^8^

The measurement of the *σ*(*E*) for Ar^+^(Ar,Ar)Ar^+^ in other QQQ instruments can be used to validate whether or not a QQQ instrument has been properly designed to be kinetically well behaved. This is essential if generic, instrument-independent CAD spectral databases are to be developed on the basis of the absolute cross sections for the CAD of *known* ionic substructures. That is, since MS/MS exploits the ion fragmentation patterns characteristic of ionic substructures, the characteristic profiles [“breakdown curves”] of ion abundance versus target thickness (or collision energy) correspond uniquely to the sequence: (parent)*_i_* $\underset{\to }{\sigma_{ij}}$ (daughter)*_j_* $\underset{\to }{\sigma_{jk}}$ (granddaughter)*_k_*, etc. Hence, computer simulation of experimentally observed breakdown curves should enable the structure of an *unknown* species to be assigned on the basis of the absolute cross sections *σ_ij_, σ_jk_*, etc., for CAD of *known* ionic substructures *i, j, k*, etc. Thus, if the calculated and experimental breakdown curves agree, the structure would be characterized. Dawson, et al. [50] demonstrated that computer simulation of breakdown curves is plausible. Hence, one can envision a CAD spectral database of critically-evaluated cross sections *σ_ij_, σ_ik_*, etc. for CAD of *known* ionic substructures measured in kinetically well-behaved instruments under standardized operating conditions. The advantages of such a database are: 1) the cross sections would uniquely characterize the CAD spectra of both known and unknown species (so long as the unknown species contain ionic substructures for which the CAD cross sections are known); 2) characterization of an unknown is *not* limited by the number of compounds in a “library”; 3) the format is compatible with its use in expert systems; and 4) end users are involved directly in its evolution by using critically-evaluated cross sections already in the database and by submitting new cross sections for inclusion in the database.

## Footnotes

2 Figures in brackets indicate literature references.

3 A “parent” ion may be a molecular radical cation, a protonated molecule, or a “progeny” fragment ion (daughter, granddaughter, etc.) produced by the CAD of a larger precursor parent ion.

4 Standard kinetic notation: [*A*^+^]_0_ and [*A*^+^] are, respectively, the intensities of the reactant ion A^+^ when measured in the absence and presence of CAD target gas; [*B*^+^] is the intensity measured for the product ion B^+^ when the target thickness is that used for the [*A*^+^] measurement; [*B*^+^]_∞_ (=[*A*^+^]_o_) is the intensity of the product ion at “infinite” reaction time and/or target thickness when all of A^+^ has been converted to B^+^.

5 It is important to note that eq (4) depends on *M*/*E.* Hence, for a given collision energy *E*, the effective target thickness *L*_eff_ [*B*] will be different for different projectile ions, and must be corrected accordingly.

6 ^x^Ar is the sum of ^40^Ar+^38^Ar+^36^Ar. The natural abundance of ^36^Ar (0.3365±0.0006% [9]) is sufficient to permit the measurement, with good signal-to-noise ratios, of the product ion growth (*Ln W* measurements).

7 Certain commercial equipment, instruments, and materials are identified in this paper in order to adequately specify the experimental procedure. In no case does such identification imply recommendation or endorsement by the National Bureau of Standards, nor does it imply that the material, instruments, or equipment identified is necessarily the best available for the purpose.

8 Interlaboratory round-robin testing of our kinetic method in various types of QQQ instruments is essential to confirm its reliability as a generic target thickness standard. Moreover, it will provide much-needed information about which QQQ instrument designs are not kinetically well behaved and therefore not well suited for the generation of standardized reference CAD spectra. The round-robin test will involve the experiment associated with figure 1(a) of [2] after first having completed in-situ target thickness calibrations of the participants’ QQQ instruments by using our kinetic method with our *σ*(*E*) for Ar^+^(Ar,Ar)Ar^+^ (this work). A test protocol is being formulated. It will address how to set *E*_coll_ and *q*_2_. Several investigators have agreed to participate. However, many more participants would be desirable to establish the degree of variability one encounters when using a standardized protocol with different operators on the same and/or different instruments of several types. Letters of inquiry from prospective participants may be sent to NBS.
